# Understanding Continuance Intention Determinants to Adopt Online Health Care Community: An Empirical Study of Food Safety

**DOI:** 10.3390/ijerph18126514

**Published:** 2021-06-17

**Authors:** Jinxin Yang, Din Jong

**Affiliations:** 1 School of Management, Hefei University of Technology, Hefei 230009, China; 2019010080@mail.hfut.edu.cn; 2 Department of Digital Design and Information Management, Chung Hwa University of Medical Technology, Tainan 717, Taiwan

**Keywords:** online healthcare community, food safety crisis, perceived critical mass, image, para-social interaction, social interaction tie, trust, partial least squares

## Abstract

The purpose of this research is to determine whether users’ social interaction tie and trust have a mediating effect on the willingness to use the online healthcare community (OHC) platform on an ongoing basis to respond to food safety crises and monitor food safety practices. During the three-month survey, we conducted an online investigation of users who had experience sharing on the OHC platform and were concerned about food safety. Thereby, three hundred and fifty-two valid questionnaires were received and partial least squares was adopted in this study to test the proposed hypotheses. The empirical results show that perceived critical mass, image, and para-social interaction strengthen the social interaction tie between users and the food safety platform. In addition, this study found that social interaction tie and trust of OHC platform users increased users’ willingness to continue using the OHC platform. This research provides OHC platform managers with an in-depth understanding of online social interactions on food safety pages. Moreover, the results of this study can help food business owners, government regulators, hospitals, and physicians to improve the way they use the Web for opinion-led food safety crises and provide insight into the intent of promoting the ongoing use of OHC platforms.

## 1. Introduction

With the increasing ease of information transmission and the continuous involvement of the Internet in the field of food safety, there is an increasing trend of food safety incidents being exposed, and food safety problems are emerging, reflecting the inadequacy of food safety supervision at this stage, and also having psychological and purchasing behavior effects on consumers. Most of the major food safety issues in recent years have been exposed by consumers through social media. In social media, every user is a watchdog and should give full play to its role as a collaborative public watchdog to make unsafe food invisible. At the same time, social media, with its low threshold of use, easy access to information, speed of dissemination, and influence in terms of breadth and depth, is attracting more and more users to participate in the attention and dissemination of food safety-related information. The rapid dissemination of food safety-related information in social media increases consumer awareness of food safety publicity and precautions, and can facilitate timely measures by regulatory authorities to solve food safety The rapid dissemination of food safety information in social media has increased consumer awareness of food safety and the need to take precautionary measures to address food safety issues, and has helped to regulate food safety issues by restraining food companies to produce and operate legally.

With the outbreak of novel coronavirus in China in February 2020, the global number of confirmed pneumonia diagnoses continues to climb, with more than 170,000 people under medical observation nationwide [1]. A large number of suspected and mildly ill patients, as well as those with outbreaks of influenza this season, are languishing in time. If they go to a hospital fever clinic, they are afraid of increasing their chances of infection; waiting in the community to be diagnosed or admitted to a hospital, the queue in front of them is even longer. As a result, a large number of patients are turning to online for help. Online medical communities are seeing peak growth. The new coronavirus epidemic has brought a huge opportunity for the accelerated development of Internet healthcare in China, and the development of the Internet healthcare industry is entering an accelerated period. As of 1 February 2021, the Ping-An Good Doctor online medical community, for example, has continued to grow at a high rate, contributing revenue of 1.566 billion yuan, up 82.4% year-on-year [2]. In Internet health, typical applications of social media in the health field, such as Online Healthcare Community (OHC), have become increasingly popular as a way to provide medical information that can help enhance and improve communication interactions between physicians and participants who care his/her health and food safety [3,4]. The OHC platform extends the communication between doctors and participants who care for their health and food safety to an online environment, so that when participants who care for their health and food safety encounter health problems, they are no longer limited to going to physical hospitals for health help, but can communicate with doctors in an online way without constraints, thus supporting the provision of maximum health information and more convenient and high-quality health The service is not only limited to going to a physical hospital for health help, but also allows participants who care for their health and food safety to communicate with doctors without the constraint of consuming the least amount of money. Information in the OHC platform spreads and evolves in real time along user relationships through publishing, forwarding, and commenting, and this information spreads in a fission-like manner. When a food safety crisis occurs, a large amount of information related to the cause of the crisis, the development situation, and the attitude of the competent authorities spreads rapidly in the network, in addition to rumor information generated by different channels. The OHC platform increases the enthusiasm and possibility of public participation in food safety crises, if it raises public awareness of food safety, better plays the role of the OHC platform in food safety regulation, promotes timely and effective measures by regulatory authorities, enhances the social responsibility of food enterprises, enables food safety problems to be solved, and enriches the results of social media information dissemination, as well as provides a basis for the monitoring and early warning of food safety public opinion on social media platforms and the construction of food safety crisis management systems [5].

When a food safety crisis event occurs, a large amount of information related to the cause of the crisis, the development situation, and the attitude of the competent authorities spreads rapidly in social media, in addition to information on rumors generated by different channels [6]. On the one hand, the release of information about food safety crises is more casual. On the online platform, everyone can be a disseminator of information, or even a creator of information, and the information released by the audience can be changed or deleted at any time. This situation has led to a lack of authority in the dissemination of information on food safety crises on online platforms, with relatively low authenticity and reliability. On the other hand, the authenticity of online media is low. The online platform allows audiences to express their views anytime and anywhere. Although relatively time-sensitive, the lack of professional theory, skills, and authoritative interpretation, as well as the lack of accountability due to virtual identities in the Internet environment, leads to less authentic information about food safety crises.

Therefore, the online statements of professionally certified doctors and well-known hospitals on the OHC platform maintain authoritative information interpretation and protect the public’s right to know. People can understand food safety crisis events more objectively through the OHC platform, adding to the public’s correct interpretation of food safety crises, and the OHC platform plays an irreplaceable role in exposing information about food safety crises. Therefore, with the advantage of accumulating resources and quick response, OHC releases objective and fair factual reports through the OHC platform to raise public awareness of food safety, better play the role of the Internet in regulating food safety, promote timely and effective measures by regulatory authorities, enhance the social responsibility of food enterprises, enable food safety issues to be resolved, and establish a bridge for consumers to disseminate information on food safety crises.

Currently, rapidly evolving social platforms are changing the way people communicate, collaborate, serve, and innovate [7,8]. Social media delves into various scenarios of application and practice, which have been extensively studied by experts and scholars, where people communicate and interact with other participants who care his/her health and food safety to obtain medical and health information through social platforms. As a result, the development of social media has driven a shift from traditional offline face-to-face consultations between patients and physicians to a virtual environment. Examples include the online medical community sites such as Patients Like Me (www.patientslikeme.com, accessed on 25 March 2021) and Good Doctor Online (www.haodf.com, accessed on 25 March 2021), where patients fully communicate with their doctors and other patients to receive more medical information.

Until empirical results are available, there is a lack of “a priori” theoretical explanations for the relationship between participants who care his/her health and food safety willingness to use OHC, and the effects of participants who care his/her health and food safety use of social media are usually based on “post hoc” analysis of survey, interview, or observational data. This paper explores theoretically the relationship between participants who care his/her health and food safety willingness to respond to food safety crises and their continued use of OHC platforms, based on “post hoc” analysis of survey, interview, or observational data. This research theoretically develops a model to evaluate the antecedents and consequences of OHC adoption by participants who care for their health and food, and which factors in the OHC model are important for participants’ willingness to continue using it. In addition, most of the previous scholars have explored the relationship between “patient–patient” or “patient–doctor”, but rarely have they integrated “patient–doctor–medical community”. “This empirical study has verified the role of doctors, hospitals, and the public in the dissemination of food safety crisis information in the OHC platform, and has enriched the research findings of public governance theory by including doctors, hospitals, and the public in the process of food safety problem solving.

Focusing on participants who care his/her health and food safety intention to use the OHC platform, this paper integrates participants who care his/her health and food safety intention to use the OHC platform by integrating social influence theory (SI) and social capital theory (SC), and empirically analyzes the impact of these two theories on intention to use. The results of the above empirical analysis suggest that the combination of these two theoretical models is able to elucidate the continued willingness to use the OHC platform for participants who care his/her health and food safety responding to food safety crises. As far as I know, there are few empirical studies in the field of food safety and social integration using the SI model and the SC theory integration model, and this paper combines these two models to fill this knowledge gap.

## 2. Literature Review

### 2.1. Social Influence Model (SIM)

The behavior of individuals is often influenced by the social environment and indirectly affects human behavior and decision making [9,10]. Venkatesh et al. [11] and other scholars integrated performance expectations with variables such as perceived usefulness, job fit, and extrinsic motivation, and unified past models and theories related to technology acceptance behavior to propose the unified theory of acceptance and use of technology (UTAUT). This is defined as the degree to which individuals expect to improve their work performance when using information technology or systems. The above study points out that social influence is one of the most important factors influencing users to accept or resist the information receiving system. The concept of social impact includes perceived critical mass and image. Chen et al. [12] proposed that these three factors of social influence have different ways of influencing the adoption of community platforms. The following discussion addresses the key factors of social influence as proposed by different scholars.

Oliver et al. [13] defined perceived critical mass as the tendency of people to follow large number of people when a certain number of participants, users, is reached. That is to say, perceived critical mass affects collective action behavior at social events. Metcalfe [14] argues that the usefulness and added value of the network are positively related to the number of users. Due to the popularity of the network, resulting in a wide range of information dissemination and influence, the more people use the network, software, games, etc., the higher their value and the more users they can attract, and when the users of the site reach a critical majority, the effectiveness generated by each additional person is even greater. Cheng et al. [15] pointed out that critical mass is an important predictor of continued intention to use because users may perceive higher value as the number of web service users continues to grow. Lynne Markus [16] found that the critical mass of interactive Internet media is quite difficult to define, and proposed the concept of perceived critical mass, which suggests that when the number of participants in a system reaches the maximum number of perceived users, the users will be willing to use it.

Boulding [17] argues that an image is defined as a perceived subjective perception and that most behaviors are guided by knowledge and information. Image has a significant impact on personal behavioral norms, as individuals react strongly not only to things but to perceived images when making decision goals, further influencing behavioral habits. The cognitive behavior of individuals in the same group or community who believe that using and accepting something new can make the individual rise in the group community, and thus increase the use of the new thing even more, has some influence on the users’ behavioral decisions, and is called image [18]. Chen et al. [19] argued that the transmission mechanism between online self-image expression and purchase intention is strengthened through perceived value, and ultimately there is a positive correlation between online self-image expression in social network games on consumers’ purchase intention. Thus, in the context of individual behavior and cognition, there is a strong link between enhancing one’s image, both in terms of external and internal factors, and community group interaction [20]. This study will be extended to the medical community platform to further explore and analyze users’ perceptions.

### 2.2. Para-Social Interaction

Horton and Wohl [21] proposed the concept of para-social interaction as a way for media providers to enable users to achieve a face-to-face-like information dissemination effect through media. The Web establishes interpersonal relationships that would otherwise require face-to-face relationships in a virtual world, building a channel of communication and connection with others, and users can participate in the interaction of virtual community networks based on common interests [22]. The Internet brings people together to share information and interact continuously to build real, genuine, and solid online relationships [23]. Members of online communities interact with each other and share ideas on the web, trying to find a space where members within a group can share social interactions and connect as users will often share behaviors or topics [24]. Kim et al. [25] used para-social interaction as a moderator of user loyalty to community online advertising and confirmed that perceived interactivity and openness have a positive and positive effect on brand loyalty to community online advertising. In a study by Lee and Gan [26], it was noted that, in users purchasing goods or services on social platforms combining SOR and PSI theories, the results showed that para-social interaction was significantly associated with impulse purchase propensity and positive affect.

### 2.3. Social Interaction Tie

Social capital is the connection between people and exists in the structure of interpersonal relationships, so the topic of social capital is complex, unmeasurable and systematized concept [27]. Shiell et al. [28] explained the relationship between social capital and health and the effectiveness of interventions to promote population health. Baum [29] and Drevdahl et al. [30] found evidence of the potential of social capital to provide new insights and pathways to improve population health and reduce health inequalities.

The structural side of social capital social interaction tie lies in the network formed by the interaction and interdependence of interpersonal relationships, which can be expressed either between people or between groups, and all of the above are a form of social system connection. A previous study by Kemperman et al. [31] analyzed the relationship between loneliness, social networks, and life circumstances in older adults, using a Bayesian belief network (BBN) modeling approach, and concluded that loneliness in older adults is directly related to personal satisfaction with social networks and satisfaction with neighborhoods. Ali et al. [32] pointed out that existing medical surveillance systems are inefficient in extracting valuable information from sensor and social network data. Thus, building a new medical surveillance framework of user-generated, unstructured medical data on social networking sites in a cloud-based environment and big data analytics engine for the accurate storage and analysis of medical data to improve classification accuracy, combining the big data analytics engine of online communities and data mining for this model can better handle heterogeneous data and improve the accuracy of health condition classification and drug side effect prediction. Block et al. [33] used a social network approach to improve the effectiveness of social distance measures and provided scientific evidence that effective social distance can effectively respond to novel coronaviruses and mitigate the negative consequences of social isolation.

### 2.4. Trust

Coleman [34] states that the concept of social capital as a resource for action is a way of introducing social structure into the rational action paradigm, when there are many specific interests in the structure of human relationships, and lasting social relationships are formed between people through trust and interaction in order for the benefits to endure. Lin and Lu [8] argue that the trust that exists in the relational side of social capital is the sum of the interpersonal relationships that develop through people’s interactions over time. In addition, Lin and Lu [8] pointed out that trust is the core of social capital. As community platforms generate interactions and behaviors among community members, such as relationships of friendship, respect and trust, which in turn influence people’s behavior and the accumulation of social capital, e.g., two people both share similar social platforms. Birkhäuer et al. [35] did an exploratory analysis with trust as the independent variable and patient satisfaction as the dependent variable and showed that trust was more correlated with patient satisfaction and less correlated with health behaviors, quality of life, and symptom severity. Tsai and Ghosha [36] examined the relationship between the structural, relational, and cognitive dimensions of social capital and the relationship between these dimensions and patterns of resource exchange and innovation within society, and noted that the structural dimension of social capital is expressed as social interaction and the relational dimension as trust. Fan and Lederman [37] pointed out that, in the OHC platform, cognitive and affective trust are more likely to make people understand the information mechanism of the platform and to make users accept the suggestions of the platform in a model based on trust theory architecture.

The prior study related to health care services and trust, it was noted that the trust distress needed by patients physically and mentally was addressed through the group wisdom of users of online health forum to understand the need to satisfy information needs, obtain emotional support and engage in social comparison [38]. Gong et al. [39] explored physician personal quality and online reputation through panel data collection based on trust theory on patient choice, concluding that the frequency of physician quality information updates and the quality of online services are equally important for online physician-patient trust. Machackova and Smahel [40] examined the role of personal factors (including demographics, online behavior, experience, and specific types of motivation) in the trustworthiness assessment of visitors to nutrition, diet, and fitness websites by examining the role that these visitors. From the above, it is clear that online health services and trust. Therefore, through this study, we could understand the importance of assessing the level of trust in online health-related information among OHC users.

## 3. Research Method

### 3.1. Theoretical Model and Hypotheses Development

When users of a community network continue to join, it not only enhances the value of that community network, but can also significantly impact the value of all users. The widespread media distribution has led to a dramatic increase in the number of community website users, which positively influences the utility value of the network and thus further influences the collective sexual behavior of the community network [41]. Thomas and Kim [42] showed that, in the new community media environment, users are offered a variety of community service sites to choose from, users can switch back and forth between community sites without monetary cost, and perceived critical mass is closely related to users’ intention to continue using. When people around are consistently using the same community website, that user will adopt the same behavior. In response to the above discussion, this study proposes Hypothesis 1:

**Hypothesis** **1** **(H1).** *Perceived critical mass has a positive effect on social interaction tie*.

Hsu and Lu [41] indicated perceived critical mass is the important factor to influence the continuance intention towards online game users. Prior research found perceived critical mass had a significant impact on the acceptance and usage of mobile health communication technologies [42]. Shen et al. [43] pointed out in his study that the advantage of social media such as instant messaging lies in the number of users, the interaction between members, and different individuals having different kinds of increases in the threshold of acceptance of innovative technology, which is first adopted by users with a lower threshold of acceptance, and then begins to spread slowly to the whole system. When it spreads to the perceived critical mass of most people, the willingness to continue using will grow rapidly.

Therefore, this study argues that online healthcare communities are also influenced by the perceived critical mass variable, meaning that when people around them are using the same online healthcare community to seek medical care, find hospitals and doctors, and discuss relevant health information with patients, individuals are likely to be further engaged with that online healthcare community platform as a result, and thus become users of that OHC, thus reaching PCM. The more easily such a result can form a consensus among users and reach an interactive behavior among them, and at the same time form an effect like a subjective norm among OHC users, which will become a characteristic of OHC and attract more similar users to join the community. In response to the above discussion, this study proposes Hypothesis 2:

**Hypothesis** **2** **(H2).** *Perceived critical mass has a positive effect on continuance intention*.

Kelman [44] stated that people will accept the influence that comes with it because they want to establish or maintain relationships with groups or other individuals. Image has a significant impact on individuals’ behavioral decisions, mainly because individuals do not react to facts when executing decisions, but to perceived impressions, which in turn influence behavior. Rogers [45] used image as a sub-construct of social capital theory, but some scholars have also found that image can facilitate the interaction of social relationships [18]. In addition to the field of research of human social activity, the idea of image is applied in the field of image research by Martineau [46], who considers the subjective attitudes, emotions, and impressions of consumers in the store’s customer base, based on the functional qualities of the store and the atmosphere of psychological feelings, as a kind of image of the store’s activity. Similar reasoning can be used to further explore and analyze the feelings of OHC platform users. Thus, this study proposes Hypothesis 3:

**Hypothesis** **3** **(H3).** *Image has a positive effect on social interaction tie*.

Zhang et al. [47] pointed out that users stop using social networking services, and the two main factors of image, external and internal factors, were used as independent variables and social interaction tie as a mediating variable to perform an exploratory analysis of users’ willingness to continue using. They finally concluded that external factors of image have a significant effect on willingness to continue using. Lee et al. [48] noted, among researchers, that, in terms of personal perceptions, there is a link between the social networking of virtual reality devices to enhance one’s image both in terms of extrinsic and intrinsic factors and the willingness to use them consistently. According to the above discussion, this study proposes Hypothesis 4:

**Hypothesis** **4** **(H4).** *Image has a positive effect on continuance intention*.

In recent years, due to the rapid development of the Internet, application software has enabled a new form of communication about food, not only strengthening the connection between existing communities and food suppliers, but also presenting the possibility to extend the relationship between communities and food consumers. With the rise of numerous OHCs, Hoerner [49] validated websites through para-social interaction, where characters and signals are constructed to stimulate computer-mediated para-social interaction. The para-social interaction perspective of Hoerner [49] considering online virtual communities was used to apply to the more interactive, social, and socially immersive OHC platform for dealing with food safety crises. In contrast to Hoerner’s [49] use of avatar facilitators, this study defines para-social interaction on the OHC platform as the facilitation of face-to-face conversations and interpersonal involvement in food safety information in a sensory or non-sensory manner through the characteristics of the medium and dialogue. Colliander and Dahlén [50] found that after para-social interaction through the interactive social function of the community platform, members communicate with each other through textual communication, exchange of information and opinions and discussion, resulting in interactive behavior. Members of the online platform share similar backgrounds, values, and interests [51]. In response to the above inferences, this study proposes Hypothesis 5:

**Hypothesis** **5** **(H5).** *Para-social interaction of OHC users has a positive effect on social interaction tie*.

Horton and Wohl [21] defined para-social interaction as the illusion of a face-to-face relationship with a media celebrity [52] and referred to the apparently seemingly face-to-face relationship between audience and performer formed by this interaction as para-social relationship. Ballantine and Martin [53] further argued that an increase in para-social relationship affects the continued use of online virtual platforms, while also pinpointing the influence of members of online virtual communities who participate in discussions on other members through para-social interaction tie theory as an analysis. Sokolova and Kefi [54] analyzed the factors influencing the success of Youtube on the ability of celebrities to influence the continued purchase intention of users and found that para-social interaction tie can successfully influence the continued use intention of users with fitness needs. Hwang and Zhang [55] pointed out the effect of online celebrity endorsed products on consumers’ intention to continue using them on social platforms, and para-social interaction tie positively influenced the online word of mouth and consumers’ intention to continue using the products. Similar to this scenario, influencers are present in all sectors: health and fitness, fashion and beauty, food, high-tech, etc. [56,57]. In OHC platform, the physicians are the influencers. OHC participants, with the OHC platform certified professional doctor’s video and blog post sharing, follow the post below the blog post, including video questions, likes, and other behavior, similar to the Instagram image or video shared by the influencers. Therefore, this study proposes Hypothesis 6:

**Hypothesis** **6** **(H6).** *Para-social interaction of OHC users has a positive effect on continuance intention*.

OHC provided a series of food safety promotion and science channels, posting content, news or events on specific food safety topics. As a result, ordinary people interested in food safety, quality of life, healthy food, and patients interested in green and organic food are attracted to interact on OHC. This type of interaction strengthens the relationship between promoters of safe food on the OHC, and this type of interpersonal interaction and communication can help users and physicians on the OHC to understand and accept common norms or practices to promote better food safety promotion and build similar values of food safety among people [58]. The interactive and communicative approach of the food discussion forum on OHC allows users to exchange information and opinions on healthy food. Since members of OHC are partly active in the virtual network with anonymous virtual identities instead of real social identities, the social network in the OHC platform is an extension of users’ real identities. Trustworthy relationships among patients, users, and physicians would increase the perceived trust via interactions in OHC. Therefore, this study proposes Hypothesis 7:

**Hypothesis** **7** **(H7).** *Social interaction tie has a positive effect on trust*.

The interconnectedness of a social system lies in the structural aspect of the composition of social capital, and its core lies in the network of interpersonal relationships formed by the interrelationships or interactions between members, both between individuals and between groups [59]. OHC is a virtual online platform that allows networking between patients, between patients and doctors, and between patients and hospitals, mainly because OHC provides communication tools (such as articles, media, videos and photos) for hospitals, doctors, patients and food providers, and each OHC user can interact and communicate with other users, maintaining and expanding the interpersonal network. Zhang et al. [60] pointed out that social capital positively influences the willingness of health professionals and general users to share knowledge in health question-and-answer (Q&A) communities and explored the factors of social capital affecting the willingness to share information in e-health settings. In response to the above inferences, this study proposes research Hypothesis 8:

**Hypothesis** **8** **(H8).** *Social interaction tie has a positive effect on continuance intention*.

Users’ trust in information systems, or lack thereof, is an important factor in the flourishing development of e-commerce and business relationships [61,62]. Yoo et al. [63] stated that important reasons for patients’ decision to use the OHC platform were related to trust, perceived information, platform reputation, and perceived physician trustworthiness, with the tendency to trust being the most important factor in changing users’ intention to adopt the OHC platform. Empirical studies of information services have also confirmed the relationship between trust and intention to use [64,65]. Therefore, trust is an effective incentive for users to use the OHC platform. In other words, if the extent to which users trust the OHC platform is strengthened, consumers are more likely to agree with the promotion of food safety on the OHC, which more strongly contributes to the reinforcement of users’ intentions regarding food safety and increases the intention of users to continue using the OHC platform. Seifert and Kwon [66] bridges the empirical gap of electronic word-of-mouth on social networks, where brand word-of-mouth and trust positively influence consumers’ purchase intentions. In response to the above inferences, this study proposes Hypothesis 9:

**Hypothesis** **9** **(H9).** *Trust has a positive effect on continuance intention*.

**Figure 1:**
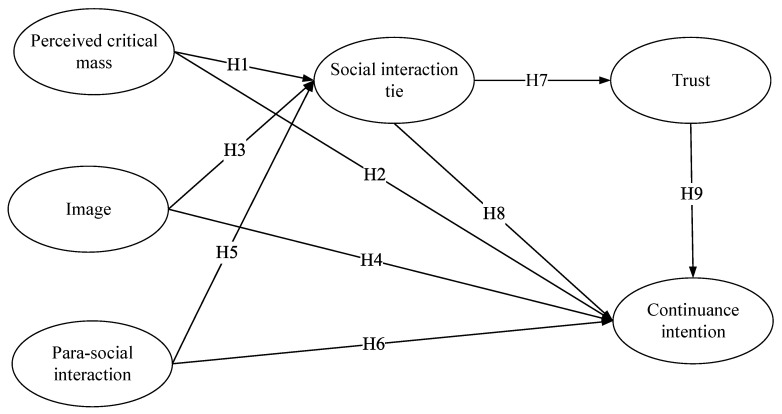
Research model.

Combining the above research hypotheses, the research model of this study is proposed (as shown in Figure 1).

### 3.2. Measurement Items and Sampling

After identifying the research topic, this study first explored the relevant literature to establish a conceptual framework of online virtual community behavior patterns and conducted case studies on relational online chatting virtual communities to identify relevant factors that influence the behavioral tendencies of online virtual communities.

The measurement variables in the study were scaled using a 7-point Likert scale ranging from strongly agree (7 points) to strongly disagree (1 point), but the behavioral measurement variables were converted to a 7-point scale from very frequent (7 points) to very infrequent (1 point) in terms of the frequency and the number of behaviors involved in responding to food safety crises on the OHC platform. The constructs used in the study and their operational definitions are shown in Table 1.

The questionnaire was conducted through the online survey of users who have shared experiences with the OHC platform and are concerned about food safety (as shown in Appendix A Table A1 and Table A2). The online web survey is produced and published through the platform (Questionnaire Star in China), and disseminated through the survey section of Dingxiang Garden Forum, health communities, WeChat and other social media platforms. The initial version of the questionnaire was checked and suggested by 4 physicians in third-class tertiary hospitals in China to meet the content validity. The formal questionnaire was distributed from 5 October 2020 to 10 January 2020, lasting 95 days. Thus, 571 questionnaires were distributed and 463 questionnaires were collected, with a recovery rate of 81.1%, among which 59 people had not heard of OHC and were not the research subjects of this paper, so they were deleted. Among the remaining 404 questionnaires, after eliminating invalid questionnaires (all the options of all the measurement items in the scale are the same and there is no differentiation) and unqualified questionnaires (the answer time is less than 50 s), 352 valid questionnaires were finally obtained, with an effective rate of 87.1%. According to Gorsuch [81], the ratio of measurement items to the number of respondents should not be less than 1:5. In this paper, there are 23 measurement items and the sample size is 352, which theoretically meets the minimum requirements to be satisfied by the study. This section focuses on descriptive statistical analysis of the demographic characteristics of the 352 samples collected and their use of OHC, with respect to the respondents’ gender, age, education, occupation, familiarity with the OHC platform, the name of the OHC they have used, and the duration of use.

Among respondents concerned about food safety using the OHC platform, in terms of gender, there were 194 men, accounting for 55.1% of the overall sample, and 158 women, accounting for 44.9%. The ratio of men to women was basically equal, but there were slightly more men than women. In terms of age, 26–30 years old accounted for the largest proportion, accounting for 29.3%, 18–25 years old accounted for the second largest proportion, accounting for 27.6%. In terms of education, the largest proportion is undergraduate, accounting for 37.5% of the total, followed by college, accounting for 26.7%. In terms of occupation, human resources and technology R&D professionals were more likely to use OHC platforms to respond to food safety crises, with 13.9% and 10.5% respectively. In terms of duration of use, users with more than 7 years and 5–7 years of internet experience used the OHC platform most frequently in response to food safety crises, at 42% and 23.6% respectively. The frequency of using OHC platforms to respond to food safety crises was highest at 28.4% on a weekly basis, followed by 18.5% on a 1–4 week basis. Good Doctor Online and DingXiang Doctor are the most familiar OHC platforms for Chinese participants who care for their health and food safety, with the majority of users using the mobile app client as their preferred tool for food safety crises.

## 4. Empirical Data Results

### 4.1. Measurement Model Analysis

In the measurement mode analysis phase, two main things are determined: (1) to verify that the measurement variables in the mode are correctly measured to their latent variables under the overall mode consideration; and (2) to check whether there are complex measurement variables loaded on different factors. The above two tests also validate two important construct validities in the research model:

- Convergent validity: The degree of correlation between different measures of variables from related variables should be high, i.e., the scores and results of measuring the same thing should be the same.
- Discriminant validity: The degree of correlation between two concepts that are not identical when measured, regardless of whether the measurers use the same method or different methods, is lower when the results are correlated.

Based on the recommendations of previous studies, the four most commonly used indicators were selected to evaluate the measurement patterns, and each indicator is described as follows:

- Individual item reliability: This index evaluates the factor loading of the latent variable by the measured variable and whether each loading is statistically significant. Table 2 shows that the factor loadings for all individual items were above 0.6 (factor loading coefficients ranged from 0.694 to 0.859), which is in accordance with the values suggested by Hair et al. [82].
- Reliability of latent variables: In order to ensure the reliability and validity of the study findings, the measurement model was tested in this study. We used three indicators, Cronbach’s alpha, composite reliability, and Rho A, to measure the reliability. The confidence of a latent variable is a component of the confidence of all its measured variables and indicates the internal consistency of the constructs. The higher the value of the confidence of a latent variable, the better the measurement indicator is able to detect the latent variable. Past studies suggested that Cronbach’s alpha, composite reliability should be above the value of 0.6 or higher and Rho A should be above 0.7 or higher (Fornell and Larcker [83]; Henseler et al. [84]). Table 3 shows that the values of the reliability indicators for each variable of the model are above the criterion of 0.7, which represents a good internal consistency of the study model.
- Average variance extracted (AVE): A higher AVE indicates higher confidence and convergent validity of the latent variable. Previous studies have suggested that the AVE value should be greater than 0.5 (Fornell and Larcker [83]; Hair et al. [82]). The information in Table 4 shows that the AVEs of the study model were higher than the proposed values for all the components (AVE values ranged from 0.589 to 0.692). From the above, it is clear that this study has convergent validity.
- This research model utilizes two ways to identify the results of discriminant validity. First, the discriminant validity is judged by whether the square root of each variable AVE is greater than the correlation coefficient between the variables. The diagonal values in Table 4 show that the minimum value of the square root of AVE is 0.767 and the maximum value of the correlation coefficient is 0.736 for each component. The comparison shows that the variables have good differential validity (Fornell and Larcker [83]). Second, as shown in Table 5, the values in the heterotrait–monotrait ratio (HTMT) of the reflective measurement model in this study ranged from 0.321 to 0.735, with each value in the matrix being less than 0.85, suggesting that the reflective measurement model in this study has discriminant validity at the conceptual level (Henseler et al. [85]).

### 4.2. Structural Model Analysis

The structural model represents the strength of the effect between variables by detecting the path coefficient (β). R-square shows the explanatory power of the dependent variable on the variance of the study model, as in the regression model. In addition, Bootstrap resampling method was used for analysis in this study. The results of the structural model analysis are shown in Table 6. Based on the entire sample, three paths are not supported (H2, H4, and H6), while the remaining six paths are all significant at the 0.001 significant level. Continuance intention is predicted by trust (beta = 0.387, *p*-value < 0.001) and social interaction tie (beta = 0.309, *p*-value < 0.001), which jointly explained 39% of the variance in continuance intention. Social interaction tie is influenced significantly by perceived critical mass (beta = 0.357, *p*-value < 0.001), image (beta = 0.266, *p*-value < 0.001), and para-social interaction (beta = 0.319, *p*-value < 0.001), with jointly 60% of the total variance explained. In that, the effect of perceived critical mass on social interaction tie is larger than image and para-social interaction.

This research further used Sobel test and Bootstrapping method with bias-corrected confidence estimates to estimate effects of the mediators [86,87]. The detection process of mediation effects was adopted from the work of Lowry and Gaskin [88] and the 95% confidence level of the specific indirect effects was obtained with 10,000 bootstrap re-samples. As shown in Table 7, three determinants (i.e., perceived critical mass, image and para-social interaction) have mediation effects on continuance intention through social interaction tie and trust.

## 5. Discussion

### 5.1. Theoretical Contribution

The main contribution of this study is the use of social interaction tie and trust from social capital theory as mediating variables and the selection of perceived critical mass and image from social influence theory to explore the factors influencing participants who care for their health and food safety willingness to use online health communities in response to food safety crises. First, we apply theories related to health care network use behavior to a scenario of participants who care his/her health and food safety responding to a food safety crisis, and propose that PCM, IM, and PSI obtained by users in the OHC platform indirectly affect ongoing use behavior, which is supported by empirical evidence.

Second, previous research on social capital theory, which treats trust as a mediating variable for a single individual, showed a significant positive effect of trust on consumers’ intention to continuous usage [27]. In contrast, this study uses two entity variables, social interaction tie and trust in social capital theory, as mediating variables, and social interaction tie and trust act as complete mediators between perceived critical mass, image, trust and continuance intention. In the OHC platform scenario, both social interaction tie and trust have a significant positive effect on consumers’ intention to continue using. When doctors on the OHC platform interact and communicate with users by posting food safety health science articles and Q&A, it will enhance the trust of users to the OHC platform. As all participants interact on the OHC platform, working together to address the food security crisis and build and maintain social capital, the expressed trust in the OHC platform and the doctors, hospitals, and other participants who care for their health and food safety on the OHC platform persists.

Third, perceived critical mass has a significant positive effect on social interaction tie. However, there was no significant effect of perceived critical mass on continuance intention. This means that more and more people use the OHC platform for health consultation, and after users reach a critical mass, each additional user will attract more users to know and use the OHC platform, which means that the OHC platform should be interactive and trusting in order to roll up the number of users more and more after users reach a critical mass. Image had a significant positive effect on social interaction tie. However, there was no significant effect of image on continuance intention. This indicates that it is the degree of improvement of the individual’s image and status in the group, participation in interactions on new topics on the OHC platform, and developing trust with other participants that can have an impact on continuance intention. Para-social interaction has a significant positive effect on social interaction tie. However, there was no significant positive effect of para-social interaction on continuance intention. This means that the OHC platform places participants with similar backgrounds, interests and values in a networked space to connect and build relationships with others, bringing them together to share topics, such as food security, and interacting consistently to build real and strong online relationships in order to have an impact on participants’ continued willingness to use it. The results show that social interaction tie and trust are the main factors that influence users’ willingness to continue using the OHC platform. The operators of OHC platforms have made it their primary goal to enhance interactive communication and to reduce information asymmetry by increasing access to medical knowledge in order to strengthen consumers’ willingness to continue using OHC platforms.

### 5.2. Practical Implication

From an economic point of view, when medical services are classified as commodities, the value of use that consumers can enjoy is positively matched with their own purchasing power, which eventually results in the elimination of winners and losers. However, pushing medical services completely to the market means that a person’s financial ability determines his or her rank of life, which not only goes against the humanitarian spirit of equality of life, but also is a retrograde step in the civilization process. Moreover, even if only from the perspective of modern economics, marketization implies information parity between buyers and sellers, in addition to considering supply and demand and price. Due to differences in education and specialization, the doctor–patient relationship is characterized by significant inequality between doctors and patients in terms of disease awareness and treatment options. This prevents patients from having the same freedom to choose products as buyers in a perfectly competitive market, and forces them to rely heavily on the expertise of their doctors and the medical standards of their providers. This paper presents basic findings on the factors that influence consumers’ willingness to continue using OHC platforms in the current healthcare market in response to the food safety crisis, as well as a specific analysis of their internal influencing mechanisms. Therefore, from the perspective of influencing factors, recommendations are made from the government side, the online healthcare community side, the consumer side, and the medical side, respectively.

Firstly, lead consumers to actively participate in OHC platform monitoring. Most previous studies have argued that a few influential individuals have an important role in the process of information dissemination and opinion formation, the significant positive effect of para-social interaction on social interaction tie in this study suggests that the proliferation of food safety crisis information in social media is driven by a large number of ordinary users in the network, confirming the findings of Newman et al. [89]. Therefore, consumers should be guided to actively participate in media. Even with food safety laws and regulations in place, food safety incidents continue to occur one after another, suggesting that food safety problems cannot be solved by mere regulation by the government ministry, and that public participation and supervision are needed. Consumers should actively participate, as soon as the production or sale of problematic food behavior immediately through the network of legitimate ways to expose or reflect to the relevant departments. For example, members in the OHC platform quickly share ways to identify problematic foods and how to save themselves in emergencies after consuming problematic foods to generate interaction and communication with other friends who share common values, and to build and maintain social capital to maintain and expand social interaction tie [90,91]. Each consumer should approach problem food from the perspective of safeguarding their own interests and the common interests of the general public so that food safety can be guaranteed.

Secondly, the OHC platform users can fully play the role of food safety supervision. The exposure of food safety crisis information by OHC platforms not only improves the transparency of information and safeguards consumer interests, but also enables a new form of communication dissemination that not only strengthens the ties of established communities, but also gives OHC platforms the possibility of extended relationships [49]. OHC users drive faster responses and problem solving from regulators and food companies through para-social interaction, and the characters and signals in the website can be constructed as a medium to stimulate attention to food safety [49].

Thirdly, OHC builds an early warning information platform for food safety. By establishing a food traceability system on the OHC platform and building a food safety early warning information platform, we are constantly improving food safety risk monitoring and encouraging consumer participation in exposing food safety issues and defending rights after crisis events. To achieve the real-time dynamic release of food safety monitoring information, special attention should be paid to the evaluation of information by the public after the release of food safety information. As food safety warning information is increasingly disseminated and more people use the OHC platform, the more valuable it will become. It will also attract more users, and when the OHC platform users reach a critical mass, the monitoring effect will be greater with each additional user to improve the positive influence of social interaction tie to other participants who care for their health and food safety [14].

In addition, food companies should engage in active communication on the OHC platform after a crisis has occurred. By looking at the way enterprises have handled food safety incidents in recent years, they have realized the impact of the change in information dissemination mode on consumers and changed their previous thinking of putting out fires by blocking, silencing and obfuscating crisis information, and most of the enterprises concerned have adopted an active communication attitude. Once a food safety issue is exposed by the media, especially by social media, it will attract a lot of attention from the general public. After the problematic food is exposed by the media, online consumers actively participate in the forwarding and discussion of the incident and the provision of evidence of the relevant food safety issues, resulting in a wide range of public opinion. Therefore, food companies should promptly respond to the problem, sincerely apologize and compensate consumers who have been harmed by their products, and take the initiative to release crisis-related information on the OHC platform instead of rushing to shirk their responsibilities. Enterprises should actively communicate with the injured consumers, media, public, government ministries and industry organizations as soon as possible after the crisis, announce the progress and treatment of the incident through OHC platform statement or press conference, and quickly pursue the legal responsibility of relevant personnel, summarize the lessons learned and take measures to avoid similar product quality problems in the future. Therefore, the perceived trust of would enhance their continuance intention towards OHC.

The OHC platform serves as an online connection platform between physicians and participants who care his/her health and food safety, and the image of physicians is closely related to the image of the platform. As online medical communities become increasingly attractive health channels, the frequency of physician quality information updates and the quality of online services have a significant impact on online physician–patient trust [38]. When a food safety crisis occurs, physicians should be the first to reassure OHC users and disseminate the right information on the OHC platform. In turn, OHC participants can alleviate the uneasiness and distress from food safety situations by satisfying information needs, gaining emotional support, and engaging in social comparison [42] supported by physicians with authority. Therefore, the information support and emotional support provided by physicians to OHC users facilitates them to build stickiness with the platform, thus encouraging trust in, and consistent use of the OHC platform.

### 5.3. Limitations and Future Work

With the rapid development of Internet technology and the growing scope of information dissemination, information technology is expanding in the health sector, and OHC platforms are becoming an important medium for responding to food safety crises. Based on the theoretical findings of this paper, we propose recommendations for the development of formalized food safety supervision and a simpler and more efficient platform management model for hospitals, as well as a foundation for the future development of “Internet + Health”. This paper focuses on the willingness to use the OHC platform in response to the food safety crisis and explores the relationship between the variables in the model. The limitations of this paper and future prospects are mainly as follows.

First, from the perspective of model construction, it is worthwhile for scholars to continue to explore and discover whether there are other factors that affect the willingness to use the OHC platform due to the specificity of the food safety crisis itself, not limited to considering PCM, IM, and PSI as independent variables. The choice of enrichment control variables can also be considered, such as studying whether differences in age, geographic region, etc. have different effects on users’ willingness to use. Secondly, when using questionnaires, attention should be paid to the breadth and diversity of the survey groups to ensure the universality of the results obtained. In addition, a variety of research methods, such as interviewing and content analysis, can be combined to make the conclusions obtained more convincing. Third, for food safety research, in terms of people of concern, it can be carried out in depth for other food and health platforms across the network, not limited to all users of the OHC platform, in order to enrich the research results.

## Figures and Tables

**Table 1:** Operational definition.

Constructs	Operational Definition	Related Documents
Perceived Critical Mass (PCM)	When the number of participants and users reaches a certain threshold, people will have a certain social tendency, i.e., a collective action that will influence this social activity.	Oliver et al. [13]; Markus et al. [67]; Hsiao et al. [68]; Lou et al. [69]; Sledgianowski et al. [70];
Image (IM)	When the OHC platform responds to food safety crisis, users’ subjective attitudes, emotions, and impressions of the OHC platform based on the functional qualities and psychological feelings of the OHC platform’s authoritative doctors who answer food safety questions scientifically and the famous hospitals who guide food safety issues positively are one of the images.	Venkatesh et al. [11]; Moore &Benbasat [18]
Para-social Interaction (PSI)	The extent to which a person-to-person conversation and interpersonal involvement is facilitated by media features and conversations in a sensory or non-sensory manner as the health visitor navigates the information in the OHC environment.	Rubin [71]; Houlberg [52]; Hoffman [72]; Giles [73]; Blanchard [22]; Ha & Jammes [74]; Ballantine and Martin [53]
Social Interaction Tie (SIT)	OHC as an online health services social platform that allows people to build interpersonal networks where doctors quickly share food safety articles, hospitals guide the public in responding to food safety crises, and participants who care his/her health and food safetysearch for safety information. Such a platform allows members of the OHC to engage in social activities among themselves, interact and communicate with other communities of interest, and build and maintain social capital.	Huang et al. [75]; Cheng and Guo [76]; Chiu et al. [77]; Okazaki et al. [78]
Trust (TR)	Trust refers to the interpersonal relationships that develop over time as people interact with each other over time. The special relationships such as friendship, respect and trust that arise from the interaction between members of online communities influence people’s behavior and the accumulation of social capital. This study concluded that hospital doctors interacting and communicating with participants who care his/her health and food safety in the food safety area on the OHC platform, and responding to food safety crises with scientific and professional health knowledge, would increase the trust in the OHC platform for users.	Chen et al. [79]
Continuance Intention (CI)	Continuance Intention refers to a situation in which an individual identifies a continuing use for an action or purpose that he or she has taken. This study defines the habit of members of the OHC platform to join and participate in this relational community, which is assessed by two attributes such as the frequency and number of times members participate in the OHC platform.	Chen et al. [79]; Chen et al. [80]

**Table 2:** Descriptive statistics and factor loadings for measurement items.

Indicator	Mean	S.D.	Skewness	Kurtosis	Factor Loading	T-Value
IM1	5.009	1.728	−0.843	−0.133	0.765	60.664
IM2	4.977	1.795	−0.759	−0.324	0.760	42.136
IM3	5.048	1.694	−0.805	−0.229	0.783	46.586
PCM1	4.807	1.816	−0.663	−0.630	0.777	33.784
PCM2	4.670	1.748	−0.573	−0.711	0.773	58.668
PCM3	4.790	1.834	−0.661	−0.672	0.786	42.416
PCM4	4.861	1.836	−0.668	−0.606	0.816	59.835
PCM5	4.540	1.987	−0.527	−0.861	0.794	44.414
PCM6	4.665	1.718	−0.518	−0.784	0.760	41.312
PI1	4.656	1.498	−0.505	−0.268	0.789	61.945
PI2	4.645	1.681	−0.550	−0.392	0.727	37.809
PI3	4.724	1.740	−0.724	−0.408	0.807	59.333
PI4	4.884	1.687	−0.764	−0.163	0.780	50.330
PI5	4.795	1.653	−0.623	−0.341	0.819	49.525
SIT1	5.543	1.488	−1.190	1.156	0.859	70.031
SIT2	5.182	1.282	−1.173	1.714	0.774	53.008
SIT3	5.443	1.482	−1.138	1.026	0.780	44.736
TR1	4.804	1.753	−0.690	−0.449	0.694	38.390
TR2	4.591	1.700	−0.582	−0.656	0.779	69.046
TR3	4.884	1.820	−0.803	−0.355	0.823	54.616
CI1	4.901	1.515	−0.648	0.256	0.857	80.583
CI2	5.082	1.640	−0.723	−0.269	0.817	74.336
CI3	4.901	1.552	−0.837	0.311	0.821	71.813

**Table 3:** Reliability and convergent validity.

	Cronbach’s Alpha	rho_A	Composite Reliability	Average Variance Extracted (AVE)
IM	0.813	0.813	0.813	0.592
PCM	0.906	0.906	0.906	0.616
PSI	0.889	0.890	0.889	0.616
SIT	0.846	0.849	0.847	0.649
TR	0.810	0.815	0.810	0.589
CI	0.871	0.871	0.871	0.692

Note: IM = Image; PCM = Perceived critical mass; PSI = Para-social interaction; SIT = Social interaction tie; TR = Trust; CI = Continuance intention.

**Table 4:** Correlation matrix.

Construct	IM	PCM	PSI	SIT	TR	CI
IM	**0.769**					
PCM	0.545	**0.785**				
PSI	0.565	0.570	**0.785**			
SIT	0.706	0.736	0.725	**0.805**		
TR	0.390	0.406	0.532	0.619	**0.767**	
CI	0.321	0.374	0.394	0.571	0.634	**0.832**

Note 1: IM = Image; PCM = Perceived critical mass; PSI = Para-social interaction; SIT = Social interaction tie; TR = Trust; CI = Continuance intention; Note 2: Diagonal elements in bold are the square root of average variance extracted (AVE) between the constructs and their indicators. Offdiagonal elements are correlations between the constructs.

**Table 5:** HTMT matrix.

Construct	IM	PCM	PSI	SIT	TR	CI
IM						
PCM	0.545					
PSI	0.566	0.571				
SIT	0.708	0.735	0.725			
TR	0.391	0.405	0.533	0.619		
CI	0.321	0.373	0.394	0.568	0.634	

Note: IM = Image; PCM = Perceived critical mass; PSI = Para-social interaction; SIT = Social interaction tie; TR = Trust; CI = Continuance intention.

**Table 6:** Path coefficients and its significances.

Path	Standardized Path Coefficient	Standard Deviation	T Statistics	*p* Values
H1: PCM -> SIT	0.357 ***	0.034	10.411	0.000
H2: PCM -> CI	0.024	0.051	0.464	0.643
H3: IM -> SIT	0.266 ***	0.042	6.396	0.000
H4: IM -> CI	−0.037	0.049	0.747	0.455
H5: PSI -> SIT	0.319 ***	0.050	6.349	0.000
H6: PSI -> CI	−0.017	0.055	0.308	0.758
H7: SIT -> TR	0.515 ***	0.047	10.954	0.000
H8: SIT -> CI	0.309 ***	0.079	3.892	0.000
H9: TR -> CI	0.387 ***	0.050	7.778	0.000

Note 1: IM = Image; PCM = Perceived critical mass; PSI = Para-social interaction; SIT = Social interaction tie; TR = Trust; CI = Continuance intention; Note 2: *** *p*-value < 0.001.

**Table 7:** Path coefficients and its significances.

Mediation Path	Z-Value of Sobel Test	Indirect Effect (IE)	Direct Effect (DE)	Mediation Type
SIT -> TR -> CI	6.564 ***	0.197 ***	0.493 ***	Partial mediation
PCM -> SIT -> TR	6.796 ***	0.321 ***	0.029	Full mediation
PCM -> SIT -> CI	6.242 ***	0.308 ***	0.025	Full mediation
IM -> SIT -> TR	7.459 ***	0.294 ***	0.025	Full mediation
IM -> SIT -> CI	6.601 ***	0.298 ***	−0.027	Full mediation
PSI -> SIT -> TR	5.574 ***	0.239 ***	0.215 **	Partial mediation
PSI -> SIT -> CI	5.999 ***	0.287 ***	0.061	Full mediation

Note 1: IM = Image; PCM = Perceived critical mass; PSI = Para-social interaction; SIT = Social interaction tie; TR = Trust; CI = Continuance intention; Note 2: ** *p*-value< 0.01; *** *p*-value< 0.001.

## Data Availability

The raw data supporting the conclusions of this article will be made available by the authors, without undue reservation, to any qualified researchers.

## Appendix A

**Table A1:** Measurement of sociodemographic variables and variables concerning information searching behavior towards OHC.

Gender	1 □Male 2 □ Female
Age	1□<18 2□18–25 3□26–30 4□31–40 5□41–50 6□51–60 7□>60
Education	1□≤junior high school 2□ junior high school (including technical secondary school) 3□ junior college 4□ undergraduate 5□ Graduate above
Which online healthcare community website do you use usually?	1□ PingAn Good Doctor 2□ChunYu Doctor 3□ Good Doctor Online 4□ One Medical website 5□ Microhospital 6□DingXiang Doctor 7□ Micropulse 8□ Ask Doctor 9□ XiaoWei healthcare 10□ others
How often do you use your favorite healthcare community website?	1□ daily 2□ 1–2 day 3□ 3–5 day 4□ 1 week 5□ 1–4 week 6□ 1–3 month 7□ 3 month above
Approximately how long do you visit your favorite healthcare community each time?	1□ Under 30 min 2□ 30 min–1 h 3□ 1–3 h 4□ 3–6 h 5□ 6 h above
Occupation	1□ Student 2□ Manufacturing 3□Sales 4□ Marketing/PR/Advertising 5□ Customer Service 6□ Administration/Logistics 7□ Human Resource 8□ Finance/Audit 9□ Civilian/Office 10□ Technician /Researchers 11□ Administrative 12□ Teachers 13□ Consultant 14□ Professionals (e.g., accountants, lawyers, architects, healthcare professionals, journalists, etc.) 15□ others
How long have you been using the Internet?	1□ daily 2□ 1–2 day 3□ 3–5 day 4□ 1 week 5□ 1–4 week 6□ 1–3 month 7□ 3 month above

**Table A2:** Questionnaire Items.

Perceived critical mass (scaling from ‘‘strongly disagree’’ to ‘‘strongly agree’’ on a seven-point scale)	
PCM1	I have bought food safety service or green food on OHC according to my friend’s advice. How do I feel about this?
PCM2	I like to buy food safety service or green food on OHC website with my family and friends.
PCM3	I go to OHC because I see family and friends using them to buy food safety services and green foods.
PCM4	On the OHC website, I always look for the doctor who answers the most questions or sends the most gifts to register and consult about food safety issues online
PCM5	On OHC website, I will only seek for food safety consultation or purchase services from doctors with many comments and high praise.
PCM6	On the OHC website, the doctors with more questions and answers, or the doctors with more gifts, the more I pay attention to them.
Image (scaling from ‘‘strongly disagree’’ to ‘‘strongly agree’’ on a seven-point scale)	
IM1	It’s an outdated phenomenon if I can’t use the OHC to seek food safety advice among my relatives and friends.
IM2	People in my relatives and friends who use the OHC have more prestige than those who do not.
IM3	People among my relatives and friends who use the OHC have a high profile.
Para-social interaction(scaling from ‘‘strongly disagree’’ to ‘‘strongly agree’’ on a seven-point scale)	
PSI1	Medical social networking sites are human when they are involved in the interaction with doctors and participants who care his/her health and food safety.
PSI2	In the process of consultation and communication on medical social networking sites, my feeling is close and there is no distance.
PSI3	If doctors and participants who care his/her health and food safety have been consulted and communicated with on medical social networks appear on other media, I will watch this program or report on it.
PSI4	Participating in the consultation sessions on medical social networking sites made me feel comfortable, as if I were with friends.
PSI5	In the process of consulting and communicating on medical social networking sites, I felt warm.
Social interaction tie (scaling from ‘‘strongly disagree’’ to ‘‘strongly agree’’ on a seven-point scale)	
SIT1	I have a high degree of interaction with members of the medical social network.
SIT2	I have spent a lot of time interacting with doctors and participants who care his/her health and food safety on medical social networking sites.
SIT3	I often communicate with doctors and participants who care his/her health and food safety on medical social networking sites.
Trust (scaling from ‘‘strongly disagree’’ to ‘‘strongly agree’’ on a seven-point scale)	
TR1	Doctors and participants who care his/her health and food safety will be willing and brave to solve the problems of other patients.
TR2	Medical social networking sites can provide reliable food safety information
TR3	In general, medical social networking sites are very trustworthy.
Continuance intention (scaling from ‘‘strongly disagree’’ to ‘‘strongly agree’’ on a seven-point scale)	
CI1	I will continue to use medical social networking sites for food safety advice or green food shopping.
CI2	In the future, I will continue to use medical social networking sites for food safety consultation or to buy green food.
CI3	I would advise my friends to use medical social networking sites for food safety advice.
